# Enhancing Pervaporation Membrane Selectivity by Incorporating Star Macromolecules Modified with Ionic Liquid for Intensification of Lactic Acid Dehydration

**DOI:** 10.3390/polym13111811

**Published:** 2021-05-31

**Authors:** Valeriia Rostovtseva, Alexandra Pulyalina, Roman Dubovenko, Ilya Faykov, Kseniya Subbotina, Natalia Saprykina, Alexander Novikov, Ludmila Vinogradova, Galina Polotskaya

**Affiliations:** 1 Institute of Chemistry, Saint Petersburg State University, 198504 Saint Petersburg, Russia; v.rostovtseva@spbu.ru (V.R.); st062444@spbu.ru (R.D.); st022544@student.spbu.ru (I.F.); subbotina.kseniya@yandex.ru (K.S.); a.s.novikov@spbu.ru (A.N.); polotskaya@hq.macro.ru (G.P.); 2 Institute of Macromolecular Compounds, Russian Academy of Sciences, 199004 Saint Petersburg, Russia; elmic@hq.macro.ru (N.S.); vinogradovalv@rambler.ru (L.V.)

**Keywords:** hybrid membrane, star macromolecule, pervaporation, ionic liquid

## Abstract

Modification of polymer matrix by hybrid fillers is a promising way to produce membranes with excellent separation efficiency due to variations in membrane structure. High-performance membranes for the pervaporation dehydration were produced by modifying poly(2,6-dimethyl-1,4-phenylene oxide) (PPO) to facilitate lactic acid purification. Ionic liquid (IL), heteroarm star macromolecules (HSM), and their combination (IL:HSM) were employed as additives to the polymer matrix. The composition and structure of hybrid membranes were characterized by X-ray diffraction and FTIR spectroscopy. Scanning electron microscopy was used to investigate the membranes surface and cross-section morphology. It was established that the inclusion of modifiers in the polymer matrix leads to the change of membrane structure. The influence of IL:HSM was also studied via sorption experiments and pervaporation of water‒lactic acid mixtures. Lactic acid is an essential compound in many industries, including food, pharmaceutical, chemical, while the recovering and purifying account for approximately 50% of its production cost. It was found that the membranes selectively remove water from the feed. Quantum mechanical calculations determine the favorable interactions between various membrane components and the liquid mixture. With IL:HSM addition, the separation factor and performance in lactic acid dehydration were improved compared with pure polymer membrane. The best performance was found for (HSM: IL)-PPO/UPM composite membrane, where the permeate flux and the separation factor of about 0.06 kg m^−2^ h^−1^ and 749, respectively, were obtained. The research results demonstrated that ionic liquids in combination with star macromolecules for membrane modification could be a promising approach for membrane design.

## 1. Introduction

Polymer film-forming materials are widely used in numerous modern technological processes using the principles of membrane separation of liquid and gas mixtures [1]. At present, polymer membranes also remain the most widely used and available materials in pervaporation (or evaporation through a membrane) processes. Still, their application, including for pervaporative dehydration of organic solvents, is limited by the trade-off between flux and selectivity, high swelling, and low mechanical stability. In that regard, the development of methods for modifying polymer films, including by mixing polymers, cross-linking, heat treatment, and modifiers, has become the main objective of recent research [2,3,4,5,6]. Hybrid membranes are developed by loading nanoparticles or other additives into a continuous polymer matrix. The combination of individual properties of polymers and fillers can lead to synergistic effects, including the formation of preferred channels for the selective transport of target compounds and the creation of barriers for undesirable molecules [7,8]. Ionic liquids have attracted attention for their use in various fields since they have such unique properties as low vapor pressure, involatility, non-combustibility, high ion conductivity, etc., and are regarded as environmentally friendly [9]. In the field of membrane technology, ionic liquids are increasingly used to modify polymer matrices in order to improve the separation properties [10,11,12,13]. Multiple attempts to load ionic liquids into pervaporation membranes have been made, including the studies on butanol recovery from aqueous mixtures [14,15,16,17] and the removal of dilute volatile organic compounds [18,19].

One of the urgent problems in separating liquid mixtures is the recovery and purification of lactic acid. Lactic acid is used in many industries, including food, pharmaceutical, chemical, and cosmetic ones. More than 90% of lactic acid produced worldwide is obtained by biological fermentation [20]. The fermentation broth contains multiple contaminants such as bacterial cells, proteins, dyes, multivalent ionic compounds, and organic acid by-products. The costs of recovering and purifying lactic acid account for approximately 50% of its production cost [21]. Considering the high optical purity of the D-lactic acid monomer required to obtain high-molecular poly (D-lactide) [22], it is crucial to choose a highly efficient, environmentally friendly, and inexpensive technology that would allow increasing the yield of lactic acid. Until recently, several methods have been used to separate and purify lactic acid, including precipitation, solvent extraction, adsorption, molecular distillation, and esterification [23,24]. However, the known methods are rather complicated and inefficient; therefore, membrane technologies as effective energy-saving methods for separating liquid mixtures have received growing attention [25]. Esterification of lactic acid used for its purification or the production of ethyl lactate has been well studied and is actively applied in industry. A wide range of research is devoted to the intensification of this process using pervaporation membrane reactors by selective removal of water from a reaction mixture and shifting the equilibrium [26,27,28,29,30,31].

However, there is only a small number of studies dedicated to the dehydration of lactic acid using membrane methods to obtain a high-purity product. There are several studies on membrane purification of lactic acid using supported liquid membrane technique [32,33,34,35]. The authors point to the efficiency of using ionic liquids as liquid carriers, including Aliquat 336, CYPHOS IL-101 [34]. M.C. Duke et al. [36] investigated molecular sieve silica (MSS) membranes for the pervaporation dehydration of lactic acid. Hydrostable CTMSS membranes showed selectivity to water and maintained high efficiency over a 300-min test period with a low flux decline. The process is also considered in work [37], along with other binary mixtures in the esterification of lactic acid with ethanol; the authors used the PERVAP^®^ 2201 membrane, which showed high permeability with low selectivity. It should be noted that sufficiently simple and effective polymer membranes for the dehydration of lactic acid have not yet been developed.

In the present work, an ionic liquid as a filler was used to obtain a membrane suitable for separating aqueous solutions of lactic acid by pervaporation.

In this work, multicomponent membranes were developed by bulk modification of PPO matrix with the novel complex fillers containing heteroarm star macromolecules (HSM) and ionic liquid (IL). HSM are the twelve-arm stars with six polystyrene arms and six arms of a diblock copolymer poly-2-vinylpyridine-*block*-poly-*tert*-butylmethacrylate covalently attached to the common fullerene (C_60_) core and IL is 1-butyl-3-methylimidazolium bis(trifluoromethylsulfonyl)imide ([BMIM][Tf_2_N]). In the previous works, we have presented a comprehensive study of the hybrid membranes containing star macromolecules with a fullerene (C_60_) branching center and 12 arms of different nature, which showed increased separation properties in the pervaporation of aqueous organic and organic mixtures [38,39,40,41]. Poly(2, 6-dimethyl-1, 4-phenylene oxide) (PPO) has been used as a polymer matrix and proved itself as a membrane material with good operational properties [42,43]. The twelve-arm stars with six polystyrene arms and six arms of a diblock copolymer poly-2-vinylpyridine-*block*-poly-*tert*-butylmethacrylate covalently attached to the common fullerene (C_60_) core had been among the promising modifiers [38,44].The transport properties of the membranes modified with complex HSM:IL filler as well as hybrid membranes HSM-PPO, IL-PPO, and polymer matrix PPO were studied in water‒lactic acid mixture separation employing vacuum pervaporation. We also paid attention to the physicochemical, XRD, and spectral characteristics of the membranes and considered the interaction of all membrane components that affect transport properties using computational methods.

## 2. Materials and Methods

### 2.1. Materials

**Figure 1:**
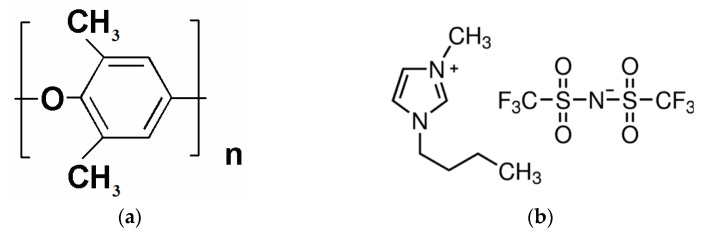
Scheme of (**a**) PPO and (**b**) IL ([BMIM][Tf_2_N]).

Poly(2,6-dimethyl-1,4-phenylene oxide) with molecular weight 172 kDa and density 1.057 g cm^−3^ (Polymer Institute, Brno, Czech Republic) was used as a membrane material (Figure 1a). Nonporous cellophane film with a thickness of 70 μm based on cellulose hydrate (Secon, GmbH, Gondelsheim, Germany) was used as a support. Chloroform was supplied by Vecton (Vecton, Saint Petersburg, Russia). Lactic acid and ionic liquid [BMIM][Tf_2_N] (Figure 1b) were purchased from Sigma-Aldrich Chemie GmbH. (Sigma-Aldrich, Schnelldorf, Germany). All the chemicals were used as received without further purification. UPM-20 (Vladipor, Vladimir, Russia) ultra-porous composite membrane based on aromatic polysulfonamide on a non-woven substrate was used as a support.

**Figure 2:**
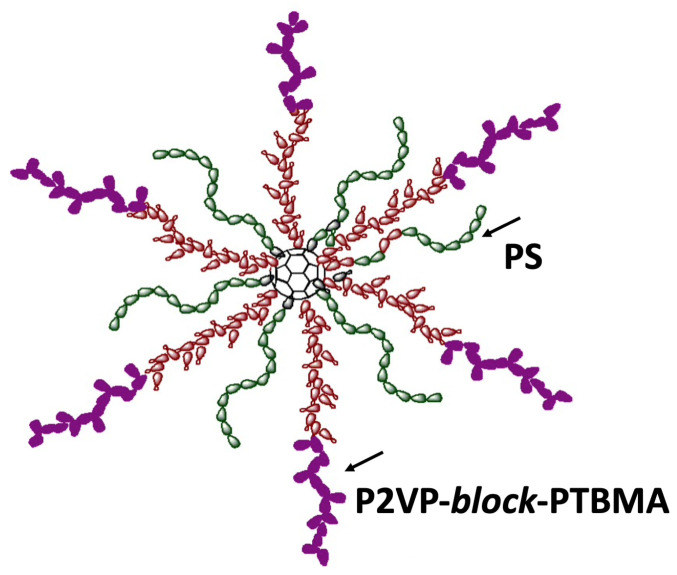
Scheme of heteroarm star macromolecules (HSM).

Star macromolecules (Figure 2) were obtained by multistage synthesis via attaching two types of polymer chains to a fullerene (C_60_) branching center using anionic polymerization methods [45]. The resultant macromolecule consists of six arms of nonpolar polystyrene (PS) and six arms of polar diblock copolymer poly-2-vinylpyridine-*block*-poly-*tert*-butylmethacrylate (P2VP–*block*-PTBMA) grafted onto a C_60_. The molecular weight of the PS arm was *M*_n_ = 6.9 kD (*M*_w_/*M*_n_ = 1.04) based on the data of size-exclusion chromatography. According to the synthesis conditions, the molecular weight of the diblock copolymer arm was two times higher than that of the PS arm at the given equal length of both block P2VP and PTBMA.

### 2.2. Membrane Preparation

The complex HSM:IL (Figure 1 and Figure 2) modifier was prepared by mixing the components in a 1:1 ratio (wt.%) in chloroform solution (concentration 2 wt.%). The resulting solution was kept under steady-state conditions for 24 h for interactions between the HSM and IL molecules. Then, the modifier solution was sonicated for 40 min.

Dense membranes based on PPO and its composites IL-PPO, HSM-PPO, or (HSM:IL)-PPO containing 5 wt.% filler were obtained by casting 2 wt.% polymeric solution in chloroform on a cellophane surface and placed on a level surface in a thermostat. The solvent was removed by evaporation at 60 °C, and the membrane was separated from the cellophane substrate and dried in a vacuum oven at 60 °C to a constant weight. The membrane thickness was ~30 µm.

**Figure 3:**
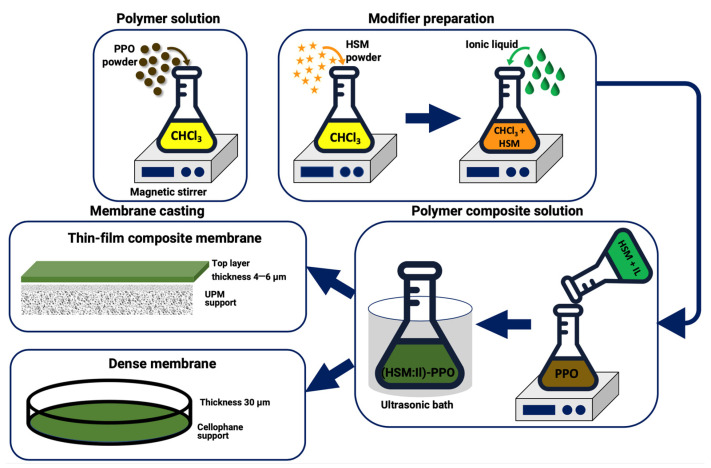
Scheme of the membrane preparation process.

Composite membranes were prepared by casting 1 wt.% PPO or (HSM:IL)-PPO solutions in chloroform on the surface of UPM support. To create a selective layer of 4–6 µm thickness, the 0.03 mL of polymer solution per 1 cm^2^ of the membrane was split. Then, the composite membrane was dried in an oven at 40 °C for 24 h and then kept in a vacuum oven at 60 °C to a constant weight. Figure 3 presents the scheme of preparation process of dense and thin film composite membranes.

### 2.3. Computational Methods

The full geometry optimization of all model structures was carried out at the HF-3c level of theory [46] with the help of the ORCA 4.2.1 program package [47]. The convergence tolerances for the geometry optimization procedure were: energy change 5.0 × 10^−6^ Eh, maximal gradient 3.0 × 10^−4^ Eh/Bohr, RMS gradient 1.0 × 10^−4^ Eh/Bohr, maximal displacement 4.0 × 10^−3^ Bohr, and RMS displacement 2.0 × 10^−3^ Bohr. The ground multiplicity state of all model systems is the singlet, and spin-restricted approximation (closed electron shell) was applied. No symmetry restrictions were applied during the geometry optimization procedure. The Hessian matrices were calculated for all optimized model structures to prove the location of correct minima on the potential energy surface (no imaginary frequencies were found in all cases). The thermodynamic parameters were calculated at 298.15 K and 1.00 atm (Tables S1 and S2, Supporting Information). The Cartesian atomic coordinates for all optimized equilibrium model structures are presented in Supporting Information as xyz-files.

### 2.4. Membrane Characterization

#### 2.4.1. Scanning Electron Microscopy (SEM)

Surface and cross-section of the membranes were examined using a Zeiss SUPRA 55VP scanning electron microscope (SEM) (Carl Zeiss, Oberkochen, Germany) equipped with In-lens SE and SE2 secondary electron detectors, a secondary electron detector for low vacuum mode (VPSE), and a four-quadrant backscattered electron detector (AsB). All samples were sputtered with a 20 nm thick carbon layer using a Quorum 150 cathode sputtering installation (Quorum Technologies Ltd., Lewes, UK) before the experiment. To observe the morphology of the cross-sections, the dried membrane samples were cracked in liquid nitrogen.

#### 2.4.2. FTIR Analysis

Functional groups’ presence and intensity were analyzed via a Bruker Tensor 27 FTIR spectrometer (Bruker Daltonics, Bremen, Germany) with a resolution of 1 cm^−1^ in the range 4000–500 cm^−1^ at 25 °C.

#### 2.4.3. X-ray Diffraction (XRD)

X-ray diffraction (XRD) analysis was performed using an X-ray diffractometer D8 DISCOVER (Bruker, Bremen, Germany) equipped with a CuKa radiation source with a wavelength of 1.54 Å. Scans were made with a step size of 0.058, ranging from 5° to 50°.

#### 2.4.4. Sorption Study

The sorption study was carried out by immersing the dense PPO membrane samples in a pure liquid at atmospheric pressure and temperature ~25 °C. At regular intervals, the samples were weighed with the analytical balance Mettler Toledo ME204 (Mettler Toledo, Columbus, OH, USA). The experiment continued until a constant weight was achieved, corresponding to a state of equilibrium.

The sorption degree (S, %) was determined as the difference between the weights of the swollen (M_s_) and dry (M_d_) membranes after the desorption experiment, referred to the weight of the dry membrane:

(1) $$S=\frac{M_{s}-M_{d}}{M_{d}}\cdot 100\%$$

### 2.5. Pervaporation

Pervaporation experiments were conducted via a lab-scale apparatus [48,49,50]. The effective membrane area was 14.8 cm^2^. Downstream pressure below 10^−2^ mm Hg was maintained on the permeate side with vacuum pump MD 1C (Vacuubrand GMBH, Wertheim, Germany), while the upstream (feed) side of the membrane was at ambient pressure. Feed composition ranged from 50 to 75 wt.% lactic acid content. The permeate was condensed in a trap cooled by liquid nitrogen. The condensed permeate was warmed up, weighed with the balance Mettler Toledo ME204 (Mettler Toledo, Columbus, OH, USA), and analyzed with a refractometer IFR–454B2M (KOMZ, Kazan, Russia) at 20 °C maintained with thermostatic bath circulator LOIP LT-411a (Loip, Saint Petersburg, Russia). The experiments were repeated at least three times, and the average value of the results was considered.

Total flux (J) was determined as the amount of liquid penetrated through the membrane area per unit time.

The separation factor β_Water/LA_ was calculated by Equation (2) as follows:

(2) $$\beta_{\mathrm{Water}/\mathrm{LA}}=\frac{Y_{\mathrm{Water}}/Y_{\mathrm{LA}}}{X_{\mathrm{Water}}/X_{\mathrm{LA}}}$$

where Y and X are the weight fraction of components in the permeate and feed, respectively.

Parameter generalizing transport properties is pervaporation separation index (PSI) calculated as:

(3) PSI=J·(β−1)

## 3. Results

### 3.1. Membrane Structure Characterization

Dense membranes based on PPO and its composites IL-PPO, HSM-PPO, and (HSM:IL)-PPO containing 5 wt.% filler were studied. The heteroarm star macromolecules (HSM) consisted of six arms of nonpolar polystyrene (PS) and six arms of polar diblock copolymer poly-2-vinylpyridine-*block*-poly-*tert*-butylmethacrylate (P2VP–*block*-PTBMA) grafted onto a C_60_ core (Figure 1) and ionic liquid (IL) 1-butyl-3-methylimidazolium bis(trifluoromethylsulfonyl)imide [BMIM][Tf_2_N] (Figure 2) were used as modifiers. Membrane structure was characterized by FTIR spectroscopy, X-ray diffraction analysis, and scanning electron microscopy.

**Figure 4:**
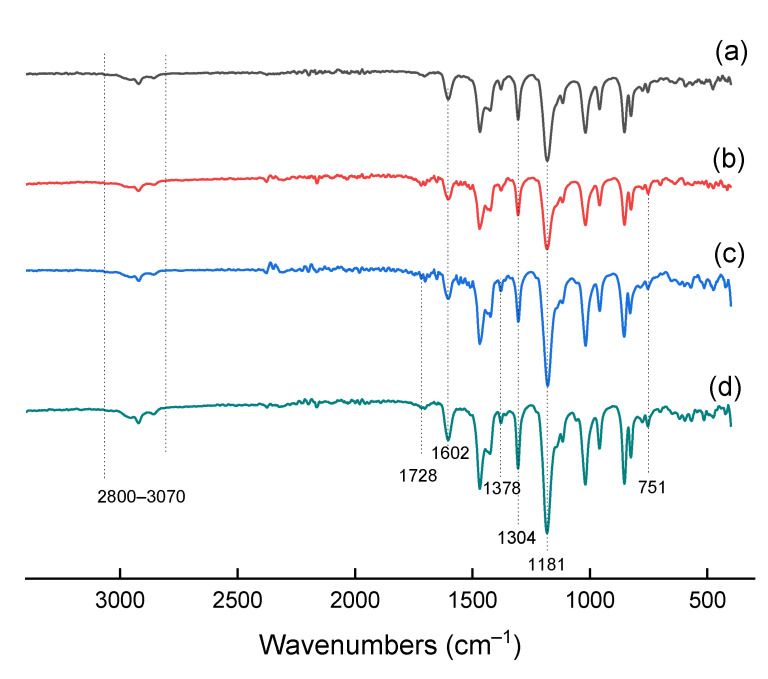
FTIR spectra of (**a**) PPO, (**b**) HSM-PPO, (**c**) IL-PPO, (**d**) (HSM:IL)-PPO.

Figure 4 shows the FTIR spectra of pristine PPO and hybrid membranes. The spectra of IL-PPO, HSM-PPO, and (HSM:IL)-PPO composites practically repeat the analogous spectra of pure PPO in the range of 500–1610 cm^−1^. The characteristic absorption bands of the phenyl group of PPO appear at approximately 1600 and 1470 cm^−1^, which corresponds to the stretching vibrations C=C and C–H of the benzene ring, respectively. Absorption at 1181 and 1304 cm^−1^ refers to symmetric and asymmetric stretching vibrations of the C–O bond. The spectra range from 2800 to 3200 cm^−1^ is the range of stretching vibrations of C–H. The bands at 3082, 3060, and 3026 cm^−1^ correspond to absorptions from stretching vibrations of aromatic C–H, while the absorption bands at 2923 and 2848 cm^−1^ originate, respectively, from asymmetric and symmetric stretching vibrations of methylene groups –CH_2_. As can be seen from the comparison of spectra 1–4 (Figure 4), when the components of the modifier are included in the PPO matrix, there are no noticeable changes in the position and intensity of the absorption bands. This may indicate only very weak interactions between the matrix polymer and modifying agents, which are not fixed due to a very low concentration of the modifier, or the absence of such interactions.

Probably, a significant factor affecting the properties of the modified membrane is the thermodynamic compatibility of PPO and the polystyrene component of HSM, which contributes to a more uniform distribution of the latter in the matrix. For composites containing N-heterocyclic and trimethylamine groups, a characteristic band appears at 1378 cm^−1^ due to the stretching C–N vibration. Two peaks are also observed: at 653 cm^−1^, associated with bending S–N–S vibrations, and at 752 cm^−1^, related to out-of-plane bending C–H vibrations in the imidazole ring. The results of IR spectroscopy indicate the successful loading of IL and HSM into the PPO polymer matrix.

**Figure 5:**
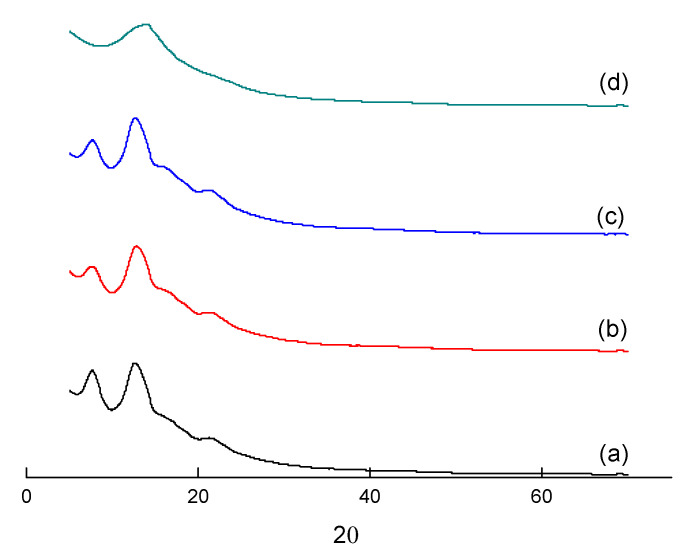
X-ray diffraction patterns of (**a**) PPO, (**b**) HSM-PPO, (**c**) (HSM:IL)-PPO, (**d**) IL-PPO membranes.

X-ray phase analysis was used to estimate the effect of the used fillers on membrane crystallinity. Figure 5 shows the XRD patterns of PPO-based membranes. It is seen that IL-PPO and (HSM:IL)-PPO membranes contain peaks indicating the presence of a certain fraction of the crystalline phase in the samples. The positions of the 2Θ reflections in the XRD patterns of the membranes containing star macromolecules (Figure 5 curve b) and star macromolecules in combination with IL (Figure 5 curve c) almost coincide with the values of 2Θ reflections for the PPO matrix polymer ((Figure 5 curve a). This indicates a similarity of these membranes in the character and degree of crystallinity. It should be noted that the XRD pattern of the IL-PPO composite is represented by a wide halo (Figure 5 curve d), reflecting the amorphous nature of the polymer, i.e., the addition of IL to PPO destroys the crystalline regions of the PPO matrix polymer. However, this effect of IL does not occur when adding the IL in combination with the star component (HSM:IL). This fact can be supported data of the previous work [51], where the XRD method was used to study PPO membranes modified with various concentrations HSM, from which it followed that the inclusion of stars in the membrane does not change the character and degree of crystallinity of the membrane.

**Figure 6:**
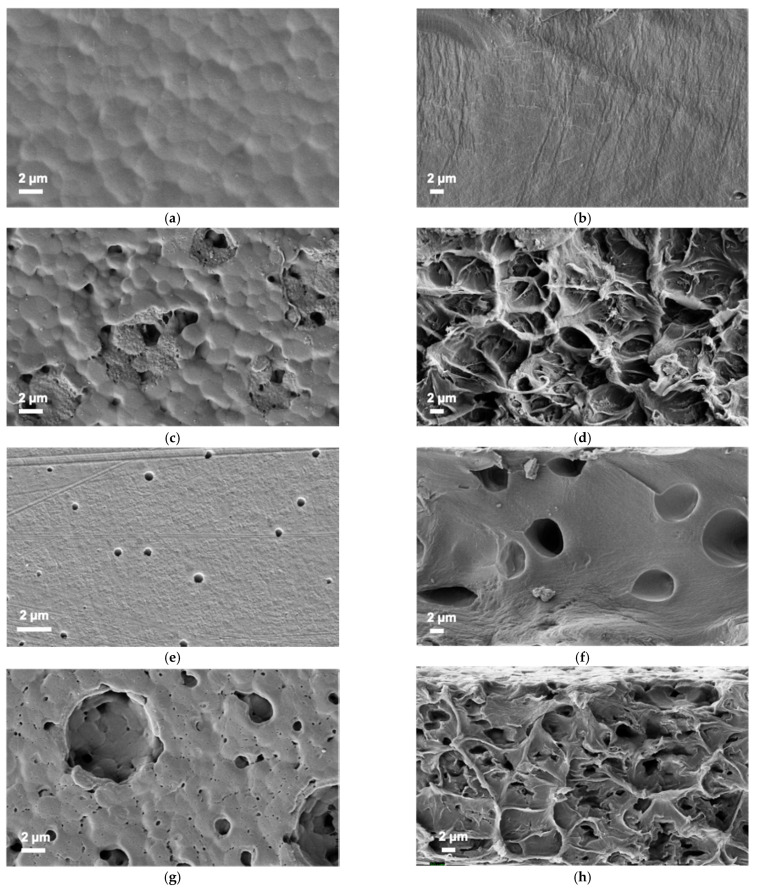
SEM micrographs of (**a**,**c**,**e**,**g**) surfaces and (**b**,**d**,**f**,**h**) cross-sections of (**a**,**b**) PPO, (**c**,**d**) HSM-PPO, (**e**,**f**) IL-PPO, (**g**,**h**) (HSM:IL)-PPO membranes.

Membrane morphology was investigated using scanning electron microscopy. Figure 6 shows SEM images of the surfaces and cross-sections of PPO, HSM-PPO, and (HSM:IL)-PPO membranes. The inclusion of fillers in the PPO matrix leads to a change of membrane surface and cross-section structure. For the HSM-PPO membrane, surface morphology varies significantly due to the formation of domain structures, which are probably formed due to the association of star macromolecules in a polymer solution and penetration of the star polar (P2VP-*block*-PTBMA) arms into the PPO matrix polymer during membrane preparation. The average domain diameter in the HSM-PPO composite is in the range of ~(1.3–5.1) μm. The images of the membrane cross-section also change. A relatively homogeneous cross-section of the matrix PPO membrane acquires the character of closed cells in the case of HSM-PPO membrane containing star macromolecules.

The IL-PPO film possess regularly arranged circular holes both on the top surface and inside the membrane (Figure 6e,f). The phase separation between PPO and IL resulted from the low solubility of IL in PPO and chloroform solution. The creation of circle domains occurs during the solvent evaporation and spontaneous upward move of IL to the top layer. This behavior is typical for hybrid membranes based on polymers and ILs [52].

The surface of the (HSM:IL)-PPO membrane looks very organized. Rounded formations with a diameter comparable to the size of domains in the HSM-PPO are observed in a rather orderly arrangement on the (HSM:IL)-PPO surface. Obviously, they arise due to the penetration of the ionic liquid into the domains, where star macromolecules play a decisive role. It should be noted that the internal structure of the (HSM:IL)-PPO membrane retains the appearance of a closed cell system.

### 3.2. Transport Properties

Mass transfer of water‒lactic acid mixture was studied to estimate transport properties of novel PPO-based membranes. The Table 1 lists some physicochemical characteristics of water and lactic acid; data on molecular weight, molar volume, radius of gyration, density, and solubility parameters can affect transport parameters of penetrants. The molecular size of lactic acid is several times higher than that of water, which can significantly affect diffusion in the process of mass transfer when separating aqueous solutions of organic acid.

The mechanism of liquid molecules transport through a dense polymer film in pervaporation can be described by the solution–diffusion model [53]. According to this model, permeability is determined by diffusion mobility of individual penetrate molecules in the membrane and solubility of the molecules in the polymer matrix.

To estimate the solubility of the separated liquids, an analysis of the solubility parameters was carried out. The Hildebrand solubility parameters given in Table 1 for liquids determine the affinity between the components of the separated mixture and the polymer. As is known [54], the smaller the difference between the solubility parameters of a polymer and a low molecular-weight component, the greater the solubility of this component in a polymer. As follows from the given data on the Hildebrand solubility parameters, lactic acid will be the most soluble both in the PPO matrix (*δ* = 18.2 (J/cm^3^)^1/2^) and in the arms of the star macromolecule, which *δ* are equal to 18.6 for PS, 18.1 for P2VP, and 16.4 for PTBMA.

**Figure 7:**
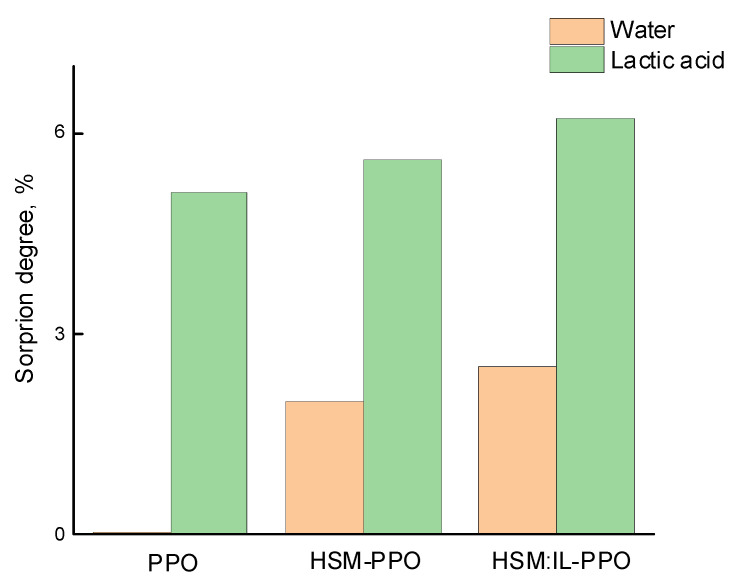
Sorption degree of water and lactic acid, 20 °C.

The equilibrium sorption degree was evaluated by carrying out sorption experiments by immersing the membrane samples in a liquid (water or lactic acid) and fixing their weight change until a constant value was reached. Figure 7 shows that the sorption degree of lactic acid is higher than that of water. As mentioned above, the matrix polymer as well as star macromolecules and ionic liquid have a greater tendency to interact with acid than with water. Since PPO has a hydrophobic nature, it almost does not sorb water. The introduction of HSM containing the polar arms with P2VP makes the membrane material more hydrophilic, which is reflected in an increase in the sorption degree of individual liquids [55].

**Figure 8:**
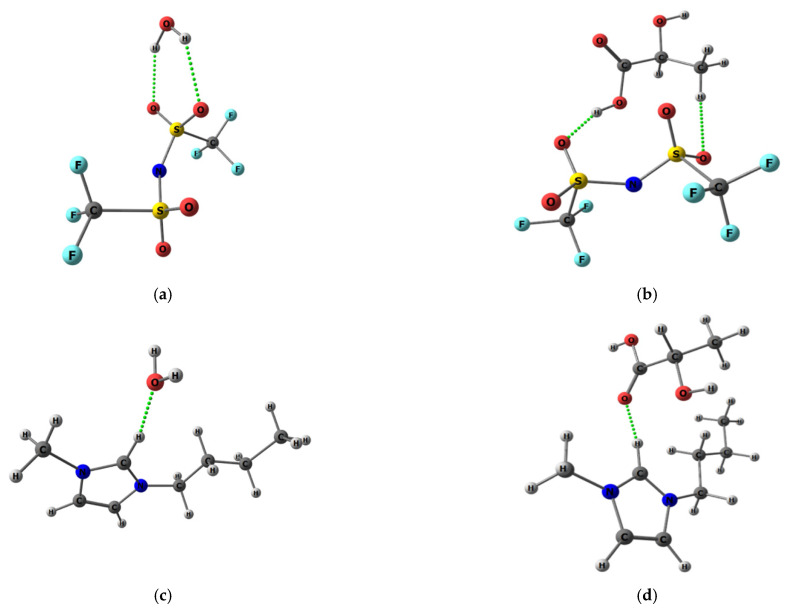
Scheme of the interaction of the anion (**a**,**b**) and cation (**c**,**d**) of IL with water (**a**,**c**) and lactic acid (**b**,**d**).

It should be noted that the ionic liquid (IL) addition to the star macromolecule also leads to a significant increase in sorption activity towards lactic acid as a result of the close values of their solubility parameters (35.1 (J/cm^3^)^1/2^ for IL and 34.1 (J/cm^3^)^1/2^ for lactic acid) due to the possibility of interaction between IL and the hydroxyl and carboxyl groups of the acid (Figure 8). However, the introduction of IL also promotes an increase in the water sorption degree in the case of (HSM:IL)-PPO as compared to HSM-PPO, since both the IL cation and anion are able to coordinate with water through hydrogen bonding, which was confirmed in the course of computer calculations.

**Figure 9:**
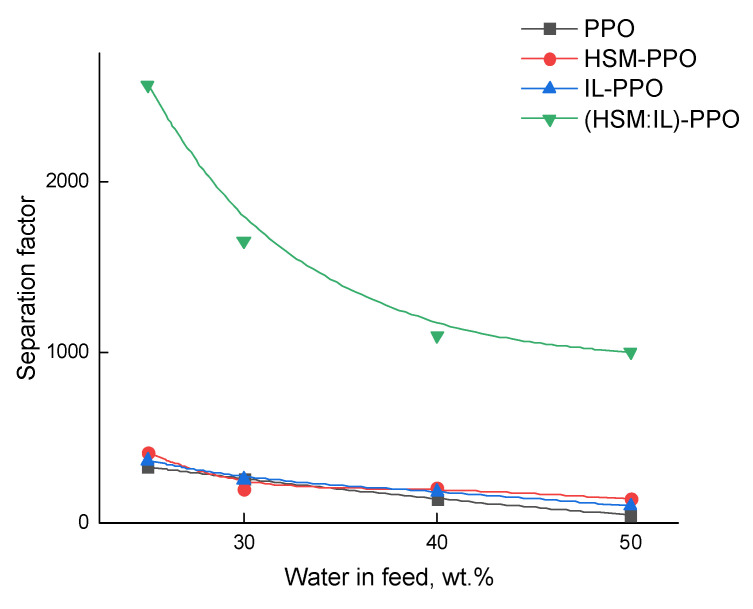
Dependence of separation factor (α_water/L.A._) on water concentration in the feed for the pervaporation of water‒lactic acid mixture, 20 °C.

**Figure 10:**
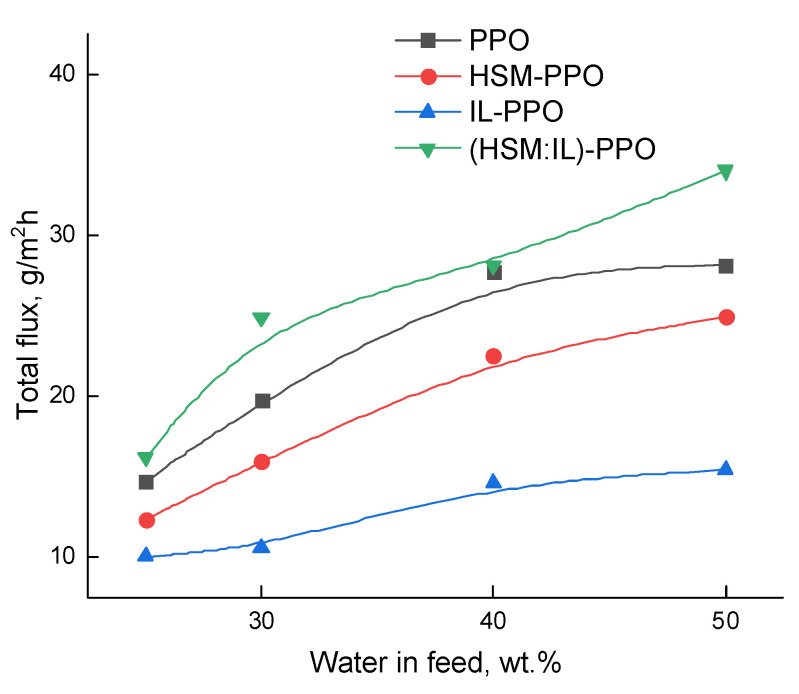
Dependences of total flux on water concentration in the feed for pervaporation of water‒lactic acid mixture, 20 °C.

Pervaporation experiments with water‒lactic acid mixture using PPO, HSM-PPO, and (HSM:IL)-PPO membranes allowed to obtain basic transport properties: total flux and separation factor (Figure 9 and Figure 10). It was found that all membranes exhibit good selectivity towards the water. The preferred water permeability is a consequence of the molecular size of penetrant molecules (Table 1): molar volume and gyration radius of water are much less than those of lactic acid. Table 1 indicates another factor affecting selectivity: the lower lactic acid vapor pressure significantly reduces the permeation driving force for this component.

Figure 9 shows the dependence of the separation factor on water concentration in the feed. For all the membranes, the separation factor decreases with increasing water content in the feed. The (HSM:IL)-PPO membrane exhibits the highest separation factor compared to others. The morphology of (HSM:IL)-PPO membranes was changed in comparison with PPO and specific zones of (HSM:IL) complex are appeared, which have increased solubility, permeability, and selectivity.

Figure 10 shows the dependences of total flux on water concentration in the feed for PPO, HSM-PPO, IL-PPO, and (HSM:IL)-PPO membranes. It can be observed that total flux increases with water content in the feed.

Increased transport properties of (HSM:IL)-PPO membrane regarding both total flux and separation factor can be associated with a change in membrane solubility. The inclusion of ionic liquid in the structure of the star macromolecule, which is the component of the (HSM: IL)-PPO membrane, increases the sorption of both water and mainly lactic acid, which determines better membrane swelling, forming and improving transport channels for the enhanced transport of smaller water molecules.

**Figure 11:**
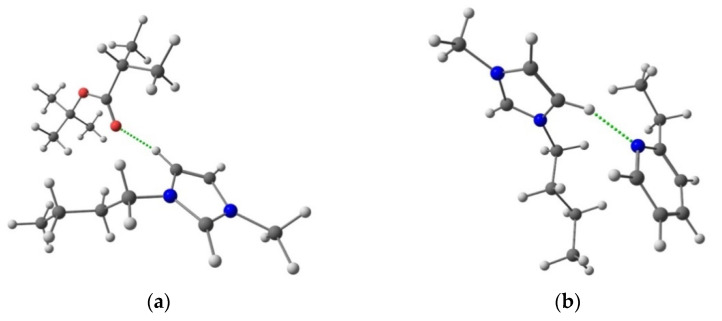
Scheme of coordination of (**a**) tert-butyl methacrylate and (**b**) 2-vinylpyridine with the IL cation.

For a more detailed investigation of the influence of modifiers on transport properties, quantum chemical calculations were used. Based on quantum chemical calculations, the heat effects of chemical reactions were obtained as the difference between the corresponding thermodynamic functions of associates and reagents. As can be seen from Table S1, when considering the interaction of IL components with the fragments of the HSM polymer chains, the coordination between IL and the star macromolecule occurs mainly due to the interaction of the IL cation and diblock copolymer (P2VP-*block*-PTBMA) (Figure 11).

When considering the heat effect of the interaction of the feed mixture components (water, lactic acid) with the polymer matrix and the modifiers, a noticeable energy gain is observed in the case of lactic acid. When considering changes in Gibbs free energy, a water molecule has a higher affinity only in the case of PS and the IL anion as compared to a lactic acid molecule, the energy gain of which is more pronounced in the case of PPO, P2VP, and the IL cation (Table S2). Meanwhile, no significant differences were found for PTBMA. It is also worth noting that, despite this comparison, the IL cation has a greater affinity for water and lactic acid than the IL anion. Comparison of the absolute values of the isobaric–isothermal potential also allows us to conclude that the interaction of the lactic acid molecules with the IL cation and the HSM component, *block*-P2VP (Figure 10), is preferable as compared to the other components of the composite.

**Figure 12:**
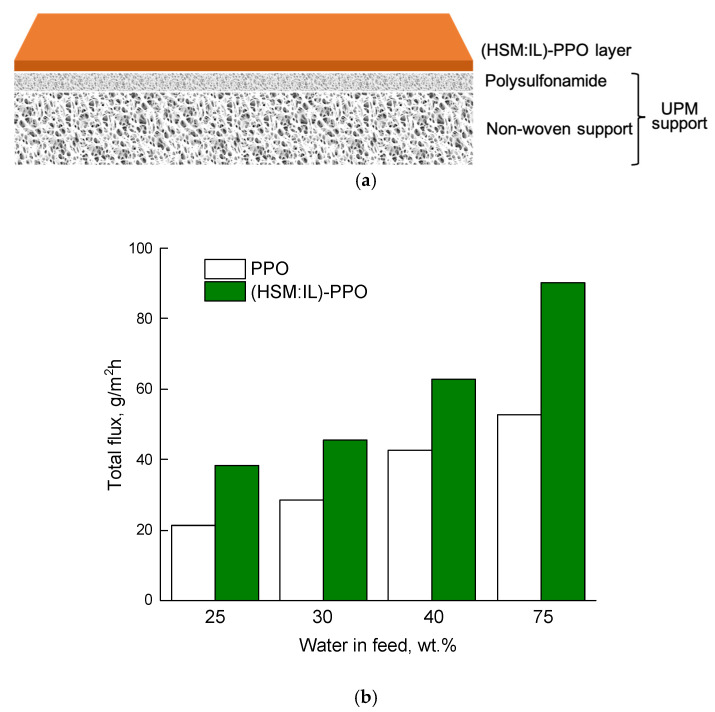
Scheme of (**a**) thin film composite membrane and (**b**) total flux through PPO/UPM and (HSM:IL)-PPO/UPM composite membranes as a function of water concentration in the feed in pervaporation of water‒lactic acid mixture, 20 °C.

The relatively low total flux is a significant disadvantage for membrane application in real processes. The total flux is determined by the thickness of the membrane that was about 30 µm for dense PPO-based films. A decrease in membrane thickness and preservation of the mechanical properties can be achieved using composite membranes. We developed a novel composite membrane consisted of a thin selective (HSM:IL)-PPO top layer on the UPM ultra porous support. Comparing the total flux through the PPO/UPM and (HSM:IL)-PPO/UPM thin film composite membrane in pervaporation of water‒lactic acid mixtures are presented in Figure 12. It is seen that the total flux of (HSM:IL)-PPO/UPM is significantly higher than in the case of the dense PPO/UPM membrane.

The transport properties of the (HSM:IL)-PPO and (HSM:IL)-PPO/UPM membranes were compared with literature data for pervaporation separation of the water−lactic acid mixture. Table 2 lists the data on total flux, water concentration in the permeate, and pervaporation separation index (*PSI*) that have been obtained for membranes under the study and commercial Pervap 2201 membrane [37]. The (HSM:IL)-PPO and (HSM:IL)-PPO/UPM membranes show higher separation efficiency in the lactic acid purification than Pervap 2201 membrane at a much lower operational temperature. Due to the higher flux of composite membrane, *PSI* of (HSM: IL)-PPO/UPM composite membrane is twice that of the (HSM:IL)-PPO thin film membrane. The data indicate the promising application of (HSM:IL)-PPO/UPM as membrane for lactic acid dehydration.

## 4. Conclusions

In this work, novel complex fillers were developed for the bulk modification of polymers as promising way of producing high performance pervaporation membranes for the intensification of lactic acid dehydration. The transport properties of poly(2,6-dimethyl-1,4-phenylene oxide) were improved by incorporating small amounts (5 wt.%) of star macromolecules modified with ionic liquid (1-butyl-3-methylimidazolium bis(trifluoromethylsulfonyl)imide). Data on the study of the physical, chemical, and transport properties of the novel membrane demonstrate the efficiency of the ionic liquid inclusion into the star macromolecule as modifier. The joint inclusion of the ionic liquid and star macromolecules in PPO leads to a change in the internal structure of membranes due to a disturbance of the initial packing of polymer chains and contributes to the formation of an ordered spatial system of transport channels. Due to the intra- and intermolecular organization of the polar chains of star macromolecules (P2VP-*block*-PTBMA) in the PPO matrix, there is a possibility to form zones of increased swelling, providing selective transport of molecules from the water‒lactic acid mixture. The high selectivity of the developed membranes with respect to water may be due to the fact that the sorption of lactic acid changes the internal structure of the walls of the transport channels, which facilitates the diffusion of water. The most thermodynamically favorable interactions between various membrane components determined by quantum chemical calculations correlate very well with the experimental results. The modified (HSM:IL)-PPO membrane demonstrates improved total flux 16.2 g/m^2^∙h and a significantly higher separation factor 2560 upon dehydration of lactic acid containing 25 wt.% water compared with the transport properties of the pure PPO membrane (14.7 g/m^2^∙h and 327, respectively). Thin-film composite membranes were successfully prepared applying commercial ultrafiltration membrane UPM-20 as support. The membrane (HSM:IL)-PPO/ UPM exhibited higher permeation flux with the same high selectivity level (99.8 wt.% water in the permeate.

## Figures and Tables

**Table 1:** Physicochemical properties of liquids.

Liquid	Molecular Weight (g/mol)	Molar Volume (cm^3^/mol)	Radius of Gyration (Å) [24]	Density (g/cm^3^) (20°)	Vapor Pressure (bar) [37]	Hildebrand Solubility Parameter δ, (J/cm^3^)^1/2^
Water	18.0	18.83	0.615	0.997	1.24 × 10^−1^	49.6
Lactic acid	90.08	88.98	3.298	1.225	1.08 × 10^−3^	34.1

**Table 2:** Comparison of membrane transport properties for the pervaporation dehydration of lactic acid.

Membrane	T, K	Water in Feed, wt.%	Total Flux, kg/m^2^ h	Water in Permeate, wt.%	PSI, kg/m^2^ h	Ref.
Pervap 2201	327	40	0.8	96.0	28.8	[37]
(HSM:IL)-PPO	293	40	0.03	99.8	31.9	Present work
(HSM:IL)-PPO/UPM	293	40	0.06	99.8	65.8	Present work

## Data Availability

Data is contained within this article.

## Supplementary Materials

The following are available online at https://www.mdpi.com/article/10.3390/polym13111811/s1, Table S1. Calculated enthalpies, entropies, and Gibbs free energies (in Hartree) for optimized equilibrium model structures (H, S, and G, respectively), Table S2. Calculated values of enthalpies and Gibbs free energies of reaction (ΔH and ΔG) for various hypothetical supramolecular association processes (in kcal/mol).
