# Supplementary material for: Enhancing Pervaporation Membrane Selectivity by Incorporating Star Macromolecules Modified with Ionic Liquid for Intensification of Lactic Acid Dehydration
Source: Polymers (Basel). 2021 May 31;13(11):1811. doi: 10.3390/polym13111811 (PMC8198700; doi:10.3390/polym13111811)
Supplement: Supplementary file 1 [file polymers-13-01811-s001.zip › polymers-1235454-supplementary.pdf]

Supporting Information

Table S1. Calculated enthalpies, entropies, and Gibbs free energies (in Hartree) for optimized equilibrium model structures (H, S, and G, respectively).

Model structure	H	S	G
H_2O---PPO	-456.46739924	0.04951203	-456.51691127
H_2O---anion_IL	-1886.23523795	0.06386289	-1886.29910085
H_2O---cation_IL	-492.93299015	0.05523530	-492.98822546
H_2O--- PS	-382.08145877	0.05090198	-382.13236075
H_2O---P2VP	-397.97620006	0.04650876	-398.02270882
H_2O--- PTBMA	-534.27067143	0.05769469	-534.32836611
PPO	-380.97628712	0.04167202	-381.01795913
anion_IL---PPO	-2191.71968742	0.07955026	-2191.79923768
anion_IL---PS	-2117.33799735	0.07836844	-2117.41636579
anion_IL---P2VP	-2133.23048400	0.07653191	-2133.30701591
anion_IL---PTBMA	-2269.52513376	0.08703361	-2269.61216737
Anion_IL	-1810.73801651	0.05687406	-1810.79489056
cation_IL---PPO	-798.40923417	0.07207407	-798.48130824
cation_IL---PS	-724.03616395	0.06694017	-724.10310413
cation_IL---P2VP	-739.93124531	0.06614001	-739.99738532
cation_IL---PTBMA	-876.22645632	0.07760167	-876.30405798
Cation_IL	-417.42924622	0.04727280	-417.47651901
PS	-306.59629603	0.03855159	-306.63484763
lactic_acid---PPO	-720.34341752	0.05995375	-720.40337128
lactic _acid---anion_IL	-2150.10491149	0.07469293	-2150.17960442
lactic acid---cation_IL	-756.80982009	0.06547369	-756.87529378
lactic_acid--- PS	-645.95426778	0.05793907	-646.01220685
lactic_acid---P2VP	-661.85148754	0.05750299	-661.90899054
lactic_acid--- PTBMA	-798.14231467	0.06744492	-798.20975959
lactic_acid	-339.35039736	0.03780734	-339.38820470
-P2VP	-322.48185358	0.03889976	-322.52075334
- PTBMA	-458.77868329	0.04943740	-458.82812069
H_2O	-75.48516676	0.02145942	-75.50662618

Table S2. Calculated values of enthalpies and Gibbs free energies of reaction (ΔH and ΔG) for various hypothetical supramolecular association processes (in kcal/mol).

Supramolecular association process	ΔH	ΔG
cation_IL + PTBMA → cation_IL---PTBMA	-11.6	0.4
cation_IL + P2VP → cation_IL---P2VP	-12.6	-0.1
cation_IL + green_PS → cation_IL---PS	-6.7	5.2
anion_IL + PTBMA → anion_IL---PTBMA	-5.3	6.8
anion_IL + red_P2VP → anion_IL---P2VP	-6.7	5.4
anion_IL + green_PS → anion_IL---PS	-2.3	8.4
cation_IL + PPO → cation_IL---PPO	-2.3	8.3
anion_IL + PPO → anion_IL---PPO	-3.4	8.5
H_2O + PPO → H_2O---PPO	-3.7	4.8
lactic_acid + PPO → lactic_acid---PPO	-10.5	1.8
H_2O + PTBMA → H_2O---PTBMA	-4.3	4.0
lactic_acid + PTBMA → lactic_acid---PTBMA	-8.3	4.1
H_2O + P2VP → H_2O---P2VP	-5.8	2.9
lactic_acid + P2VP → lactic_acid---P2VP	-12.1	0.0
H_2O + PS → H_2O---PS	0.0	5.7
lactic_acid + PS → lactic_acid--- PS.	-4.8	6.8
H_2O + cation_IL → H_2O---cation_IL	-11.7	-3.2
lactic_acid + cation_IL → lactic_acid---cation_IL	-18.9	-6.6
H_2O + anion_IL → H_2O---anion_IL	-7.6	1.5
lactic_acid + anion_IL → lactic_acid---anion_IL	-10.4	2.2
